# Occipital neuralgia secondary to CerebroSpinal fluid leak

**DOI:** 10.1186/1129-2377-14-S1-P151

**Published:** 2013-02-21

**Authors:** H Ansari

**Affiliations:** 1 Mayo Clinic, USA

## Introduction

Spontaneous CSF leakage is now recognized as a secondary chronic daily headache (CDH) and its characteristic feature is a postural (orthostatic) headache. Despite occipital headaches being common, only one published case report exists of occipital neuralgia associated with significant cerebral descent from CSF leakage. Our case is the first one, occurring without prominent cerebral descent. Potential pathogenic mechanisms are discussed.

## Purpose

To report a spontaneous CSF leak as an unusual cause for occipital neuralgia.

## Methods

Case report.

## Results

A 51-year-old woman with CDH for 3 years presented with bilateral, pressure-like headache aggravated by valsalva and alleviated by lying down. Neurologic exam, brain MRI/MRV/MRA and cervical MRI were all unremarkable. Considering a New Daily Persistent Headache (NDPH), amitriptyline was started but failed. Her headache changed to bilateral occipital, shock-like pains, lasting seconds to minutes superimposed on a “spongy, numb” occipital sensation but without postural component. Occipital nerve block resulted in a dramatic response. Repeat brain MRI showed dural enhancement with subtle “brain sagging”. An empiric lumbar epidural blood patch failed. Spinal MRI showed extradural fluid collections in the lower thoracic region. Conventional computed Tomography (CT) myelogram confirmed a mid-thoracic CSF leak. Subsequent dynamic CT myelography confirmed the location of fast CSF leakage at T4-T5 secondary to a disc extrusion. A decompressive laminectomy with repair of the CSF leak completely aborted her headaches.

## Conclusions

Occipital neuralgia secondary to CSF leakage may be an under-diagnosed phenomenon. Pressure to the C2-C3 nerve roots during prominent cerebral descent may be the underlying headache cause. In cases without significant cerebral descent such as ours, traction of the C2-C3 nerve roots or the occipital nerve may be a better explanation. Other mechanisms, however, are likely role players as occipital neuralgia is a rare happening in the setting of a spontaneous CSF leak.
